# Left atrial strain on 2D-STE in pediatric dilated cardiomyopathy

**DOI:** 10.1186/s44156-026-00126-2

**Published:** 2026-07-06

**Authors:** Yi Wu, Qiu-Qin Xu, Wei Liu, Cui Hou, Hui Wang, Jie Shen, Yao-Wen Sun, Pei-Pei Gu, Xue-Ying Si, Hai-Tao Lv, Ling Sun, Yue-Yue Ding

**Affiliations:** 1 Department of Ultrasonography, Jing’an District Centre Hospital of Shanghai, Shanghai, 200040 China; 2 Department of Cardiology, Children’s Hospital of Soochow University, Zhongnan Street 92, Suzhou, Jiangsu Province 215025 China

**Keywords:** Dilated cardiomyopathy, Echocardiography, Left atrial strain, Two-dimensional speckle tracking echocardiography

## Abstract

**Objective:**

To characterize the left atrial (LA) strain of pediatric dilated cardiomyopathy (DCM) on two-dimensional speckle tracking echocardiography (2D-STE), and elucidate the progression of LA strain with long-term medical therapy in such patients.

**Methods:**

Children with DCM who received standardized medical treatment for > 1 year at Children’s Hospital of Soochow University between January 2018 – January 2021 were retrospectively reviewed. Age- and sex-matched patients with normal echocardiography were included as controls. Routine echocardiography was performed. 2D-STE was applied to obtain LA strain and tissue mitral annular displacement (TMAD) parameters. Echocardiographic images at baseline, 1-year post-treatment (Period 1), and > 2-year post-treatment (Period 2) were analyzed.

**Results:**

A total of 33 DCM patients were included, among whom 19 had isolated DCM, while 14 had DCM associated with left ventricular (LV) hypertrabeculation. At baseline, the age range of these children was 1 month to 144 months, and significantly lower left ventricular ejection fraction (LVEF) and fractional shortening (LVFS) (*P* < 0.05) were observed among the patients compared to controls. Among the LA strain parameters, significantly lower reservoir strain (LASr) and conduit strain (LAScd) (*P* < 0.05) were also demonstrated. And significantly higher LVEF, LVFS, LASr and LAScd and significantly lower IVSd-Z and LVEDd-Z were observed in DCM associated with LV hypertrabeculation patients compared to isolated DCM patients. Between Period 1 and 2, significant increase in LVEF, LVFS, LASr, and LAScd were observed (*P* < 0.05).

**Conclusions:**

Following long-term standardized medication treatment, children with DCM exhibited significant improvements in left atrial reservoir and conduit functions. LAScd carries the potential as a sensitive imaging modality for LA strain in pediatric DCM.

**Supplementary Information:**

The online version contains supplementary material available at 10.1186/s44156-026-00126-2.

## Background

Cardiomyopathy is mainly characterized by structurally and functionally abnormal myocytes and not directly related to cardiovascular diseases such as coronary artery disease (CAD), hypertension, valvular disease, and congenital heart disease (CHD). Recent consensus classifications have defined pediatric cardiomyopathies as dilated cardiomyopathy (DCM), hypertrophic cardiomyopathy (HCM), restrictive cardiomyopathy (RCM), non-dilated left ventricular cardiomyopathy (NDLVC), and arrhythmogenic right ventricular cardiomyopathy (ARVC) [1]. LV hypertrabeculation is currently considered to be a phenotypic trait that can occur either in isolation or in association with other cardiomyopathies [1].

There are international studies with quite different distribution for prevalence of cardiomyopathies. The annual incidence of DCM has been estimated at 5–8 cases per 100 000 people [2], but this was thought to be an underestimate due to incomplete ascertainment. In the United States, Finland, and Australia, the overall incidence of primary cardiomyopathies among children < 20 years of age has been reported to be approximately 1 in 100,000 persons per year, with a peak incidence observed among those ≤ 2 years of age [3, 4]. In China, although there is no exact incidence, a 12-year multicenter study involving 33 hospitals found that children hospitalized with primary cardiomyopathy accounted for 0.079% of all pediatric inpatient admissions during the same period, with DCM being the most prevalent subtype (32.95%). Moreover, the total number of annual hospitalizations of children with cardiomyopathy has been increasing yearly [5]. The prognosis for children diagnosed with pediatric DCM is grim, with nearly 40% undergoing heart transplantation or dying within two years of diagnosis [6].

In the last decade, increasing interest has been placed in the structural and functional assessment of the left atrium for the diagnosis and prediction of cardiac dysfunction. Left atrial (LA) function can be categorized into three main components — reservoir function, conduit function, and contraction function [7]. Studies have shown that LA function closely reflects both the diastolic and systolic function of the left ventricle, and has been considered a biomarker for adverse cardiovascular events in heart failure with preserved ejection fraction [8–11]. Evaluating LA function in pediatric patients remains challenging [12]. LA functional parameters derived from adult studies may be insufficiently sensitive among the pediatric population [13]. It is necessary to assess the LA function of pediatric cardiomyopathies.

Two-dimensional speckle-tracking echocardiography (2D-STE) has become an established method for quantifying myocardial function [14–16]. 2D-STE involves tracking the motion of spots within the myocardium throughout the cardiac cycle for the evaluation of parameters such as myocardial velocity, displacement, and strain. This imaging technique is load- and morphology-independent, and is not influenced by sampling angle. Moreover, evidence has confirmed that atrial strain evaluation using 2D-STE is well-validated in both adults and children [17, 18].

Diastolic function in children is very difficult to accurately measure, especially in the early stages. LA strain is an easy to perform, highly reproducible echocardiographic measurement of diastolic function that has been shown to demonstrate pre-clinical diastolic function in children with chronic kidney disease, type 2 diabetes mellitus et al. and with good invasive correlation with catheter measured LVED pressure [12]. We thereby aimed to characterize the LA strain of pediatric DCM patients using 2D-STE, and evaluate the role of this imaging modality in the treatment response monitoring of such patients.

## Methods

### Study population

Children diagnosed with DCM admitted to the Children’s Hospital of Soochow University between January 2018 – January 2021 were retrospectively reviewed. Echocardiographic parameters were analyzed for these children at initial assessment (Baseline), 1 year (Period 1) and more than 2 years after treatment (Period 2). The exclusion criteria involved (1) the abnormal loading conditions such as concurrent hypertension, other congenital heart diseases, valvular disease, or ischemic heart disease and (2) not previously treated, or received < 1 year of treatment at our institute.

DCM was defined as a left ventricular end-diastolic dimension Z score (LVEDd-Z) of > 2, a left ventricular ejection fraction (LVEF) of < 55%, and a left ventricular fractional shortening (LVFS) of < 28% [19]. It was divided into two subtypes according to whether it was combined with LV hypertrabeculation: isolated DCM and associated with LV hypertrabeculation. LV hypertrabeculation was diagnosed according to the echocardiographic criteria proposed by Jenni et al. [20, 21], which included the presence of a severely thickened, two-layered myocardium, and a non-compacted subendocardial-to-compacted subepicardial wall thickness ratio of > 2:1 at end-systole in the short-axis view.

Given the significant differences in indicators such as age, BSA, BMI, and gender between the Baseline group and Group 2, two separate control groups each consisting of 30 healthy children were established, designated as Control Group 1 and Control Group 2, respectively. Echocardiography was indicated in such patients for either the investigation of a heart murmur, or a general health checkup.

The study was approved by the Ethics Committee of the hospital, who waived the need for written informed consent from the legal guardians or next of kin of the patients.

### Instrumentation

Imaging was performed using a color Doppler ultrasound device (EPIQ-7 C; Philips, Amsterdam, the Netherlands) equipped with a S5-1 (3.5–5.0 MHz) or S8-3 (4–7.5 MHz) probe according to guidelines by the American Society of Echocardiography and the European Association of Cardiovascular Imaging [22, 23]. Conventional LA and LV parameters such as wall thickness and cavity dimension at end diastole, and diastolic function were collected. Wall thickness and cavity dimensions were calculated using the Z-score regression equations proposed by Wang et al. [24]. In addition, LVEF was evaluated using the biplane Simpson’s method.

LA strain analysis was performed using an automated offline speckle tracking echocardiography software (AutoStrain LA, QLab 13.0, EPIQ-7 C, Philips, Amsterdam, the Netherlands). The region of interest was automatically generated by delineation of the epicardial and endocardial borders. When the automatic measurement of LA strain was inaccurate, manual editing of the LA borders was performed at end-systole or end-diastole to improve accuracy. The measured parameters included reservoir strain, conduit strain, and contraction strain in end diastole (LASr, LAScd and LASct, respectively) [25] (Supplemental Fig. 1A, 1B). Left ventricular global longitudinal strain (GLS) was also carried out using the same method.

Tissue mitral annular displacement (TMAD) was assessed using the automated cardiac motion quantification software (QLab 13.0, EPIQ-7 C, Philips, Amsterdam, the Netherlands). The measured parameters included the mean displacement of the mitral annulus (TMAD AP4 Midpt, TMAD AP2 Midpt), and the mean displacement rate of the mitral annulus (TMAD AP4 Midpt%, TMAD AP2 Midpt%) (Supplemental Fig. 1C, 1D).

All imaging analyses were performed by 2 sonographers with 10- and 12-year experience, respectively. Echocardiographic parameters were analyzed for these children at initial assessment (Baseline), 1 year post-treatment (Period 1), and > 2 years post-treatment (Period 2). The mean value of 3 readings was recorded for all measured parameters.

### Statistical analyses

Statistical analyses were performed using SPSS 26.0 (IBM, Armonk, New York, USA). Continuous variables are expressed as mean ± standard deviation (SD), while categorical variables are expressed as number and percentage. The student’s *t*-test and Wilcoxon test were used for comparison of normally and non-normally distributed data, respectively. The repeated measures ANOVA and Friedman test were used for multigroup comparisons. Association was evaluated using Pearson correlation coefficient. Inter- and intra-observer variabilities were assessed using Bland-Altman analysis. Statistical significance was defined as *P* < 0.05.

### Drug treatments

The treatment regimens of each patient were formulated by senior pediatric cardiologists, with dosages adjusted where necessary. The principle of therapy was to improve heart function, with commonly used drugs including angiotensin-converting enzyme inhibitors (ACEIs), β-blockers, diuretics, positive inotropes, and metabolic modulators [26]. The list of medications used, and their standard dosages, are shown in Supplemental Table 1. In general, positive inotropes were indicated among patients with reduced ejection fraction, digitalis preparations were prescribed as rate control for arrhythmias, while phosphodiesterase inhibitors were administered in the presence of pulmonary hypertension.

### Repeatability and reproducibility of LASr and LAScd

All inter-and intra-observer variability results for LASr and LAScd were shown in Supplemental Table 4. Repeatability and reproducibility of both echocardiographic measures were deemed favorable (Supplemental Fig. 2, Supplemental Table 2).

## Results

### General information of the DCM patients

**Fig. 1:**
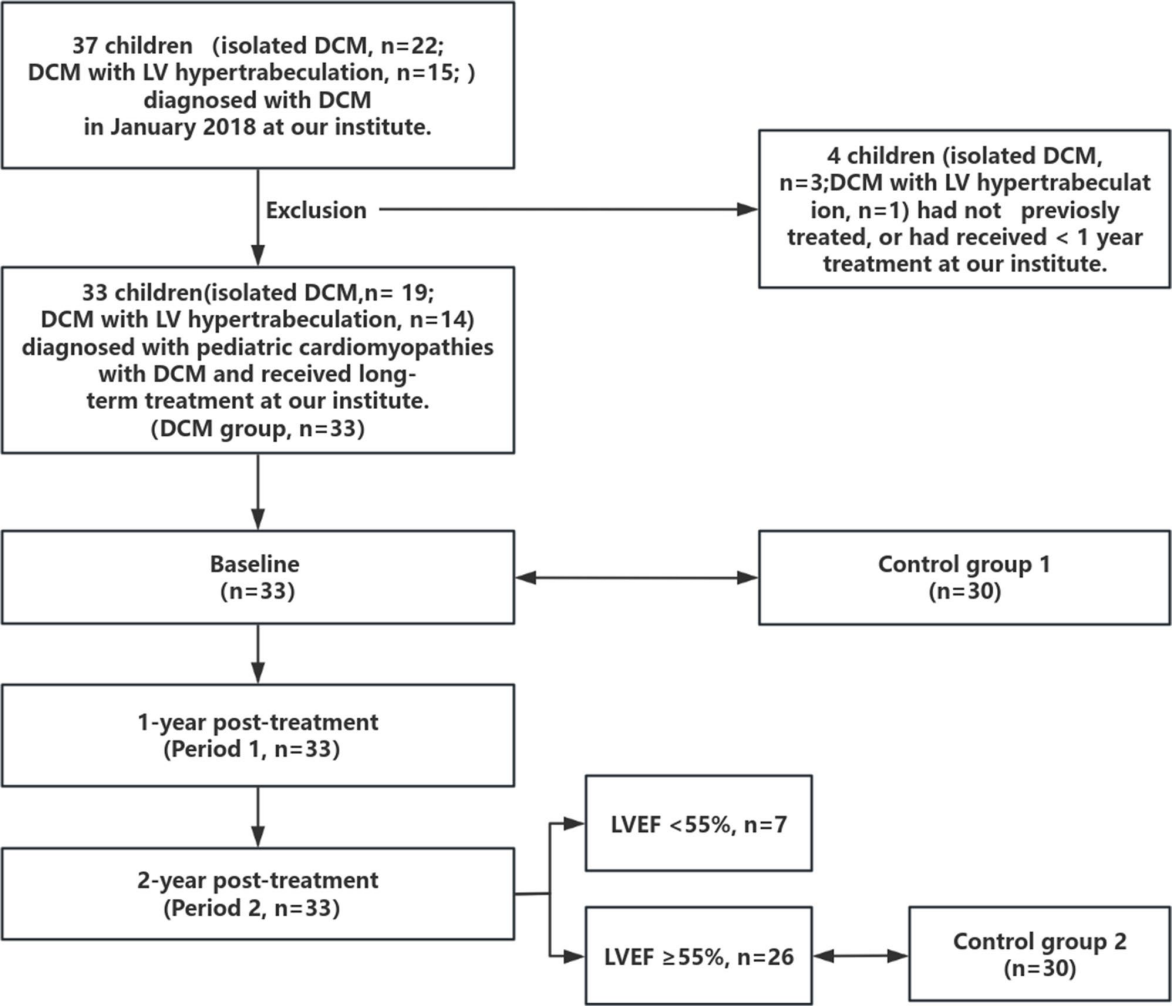
The flowchart of this retrospectively study. Among the 37 DCM patients (isolated DCM, *n* = 22; DCM with LV hypertrabeculation, *n* = 15), 4 were excluded due to lack of or < 1-year duration of treatment at our institute (isolated DCM, *n* = 3; DCM with LV hypertrabeculation, *n* = 1). Eventually, 33 DCM patients were included, of whom 19 and 14 had isolated DCM and DCM associated with LV hypertrabeculation, respectively. Echocardiographic parameters were analyzed for these children at initial assessment (Baseline), 1 year (Period 1) and more than 2 years after treatment (Period 2). 30 healthy children matched for the condition of the DCM group at baseline were selected as Control Group 1, while another 30 healthy children matched for the condition of patients whose LVEF ≥ 55% and LVFS ≥ 28% in Period 2 were selected as Control Group 2

Among the 37 hospitalized patients (isolated DCM, *n* = 22; DCM associated with LV hypertrabeculation, *n* = 15), 4 patients (isolated DCM, *n* = 3; DCM associated with LV hypertrabeculation, *n* = 1) were excluded due to lack of or < 1-year duration of treatment at our institute. Eventually, 33 patients were included, of whom 19 and 14 had isolated DCM and DCM associated with LV hypertrabeculation, respectively (Fig. 1).

Among them, 16 (48.5%) and 17 (51.5%) were male and female, respectively. The median age was 14 months (range, 7–48 months). The median duration of treatment was 26 months (range, 24–32 months). In terms of etiology, the genetic testing had discovered that 10 (30.3%) were genetic, 4 (12.1%) were inflammatory, 1 (3.0%) was autoimmunity, while 18 (54.5%) were unknown. No deaths occurred during the study period. The baseline characteristics of the included patients were summarized in Table 1. In addition, there were 2 children classified as NYHA class I, 15 as NYHA class II, 16 as NYHA class III, and 0 as NYHA class IV in Table 1.

**Table 1:** Clinical characteristics of the DCM patients (*n* = 33)

Variables	Numbers
**Sex**, male/female	16/17
**Age**, months (Interquartile range, IQR)	14 (7, 48)
**BMI**, kg/m^2^ (IQR)	15.19 (13.97, 16.28)
**Follow-up duration** (IQR)	26 (24, 32)
**Cardiomyopathy type**	
Isolated DCM, n (%)	19 (57.6)
DCM associated with LV hypertrabeculation, n (%)	14 (42.4)
**Etiology**	
Genetic, n (%)	10 (30.3)
Inflammatory, n (%)	4 (12.1)
Autoimmunity, n (%)	1 (3.0)
Unknown, n (%)	18 (54.5)
**NYHA classification**	
NYHA class I, n (%)	2 (6.0)
NYHA class II, n (%)	15 (45.5)
NYHA class III, n (%)	16 (48.5)
NYHA class IV, n (%)	0 (0)

### Echocardiographic parameters between DCM patients at baseline period and Control Group 1

**Fig. 2:**
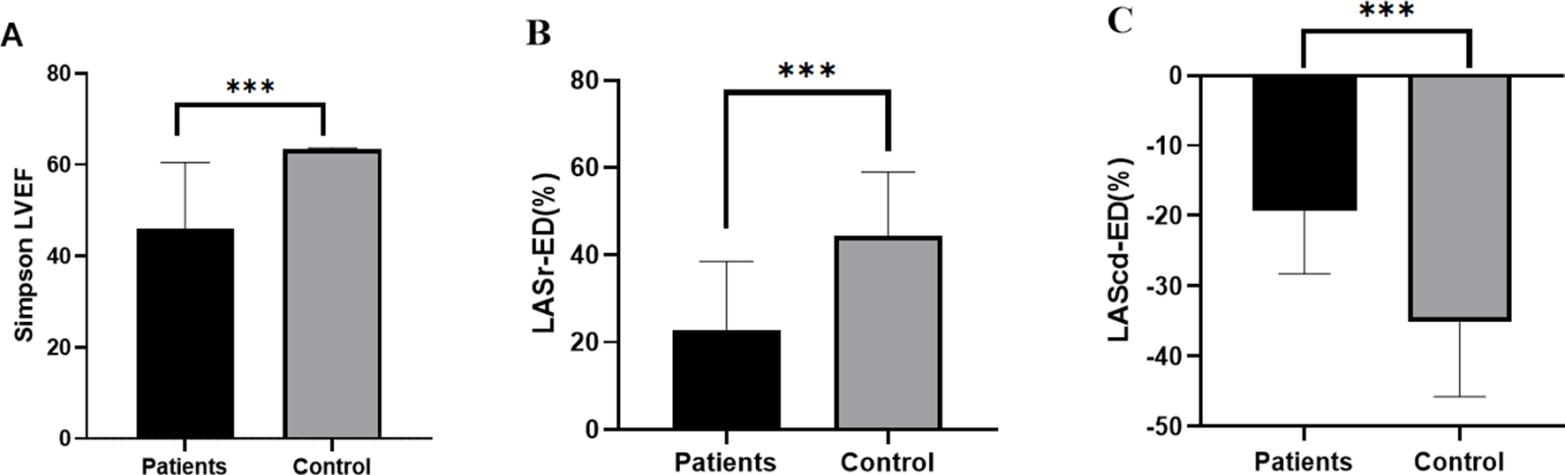
Conventional echocardiographic and LA strain parameters between DCM patients at baseline period and Control Group 1. Significant differences were observed in (**A**) LVEF, (**B**) LA reservoir strain at end diastole, and (**C**) LA conduit strain at end diastole. *** *P* value < 0.001

We compared DCM patients with Control Group 1 in order to define the extent of left heart functions impairment without treatment at baseline period. Significantly lower LVEF, LVFS, E peak, e’ peak, LASr, LAScd, GLS indicators and higher IVSd-Z, LVEDd-Z, LVPWd-Z, LA-Z, E/e’ ratio values were observed (*P* < 0.05). There were no significances in indicators such as E/A, LASct, and all TMAD between these two groups. (Table 2; Fig. 2).

**Table 2:** Echocardiographic features between patients and Controls Group 1

Variable	Patients (*n* = 33)	Control Group 1 (*n* = 30)	*P* value
**Sex**,** male/female**	16/17	12/18	NS
**Age**,** months**	14 (7, 48)	16 (10, 48)	NS
**BMI**,** kg/m**^**2**^	15.19 (13.97, 16.28)	16.03 (15.28, 17.09)	NS
**LV systolic function**			
LVEF, %	49.00 (29.25, 59.50)	62.00 (60.25, 65.75)	0.000^***^
LVFS, %	26.50 (16.25, 26.50)	38.50 (36.25, 41.00)	0.000^***^
IVSd-Z	1.75 (1.23, 3.33)	0.25 (-0.48, 1.85)	0.000^***^
LVEDd-Z	4.93 ± 2.35	0.13 ± 0.68	0.000^***^
LVPWd-Z	2.33 ± 0.81	0.25 ± 0.92	0.000^***^
LA-Z	0.79 ± 1.67	-0.88 ± 0.89	0.016^*^
GLS, %	-11.07 ± 3.38	-21.58 ± 0.94	0.000^***^
**LV diastolic function**			
E peak, cm/s	100.00 (98.00, 110.00)	110.00 (100.00, 118.00)	0.015^*^
e’ peak, cm/s	7.41 ± 1.56	11.87 ± 1.60	0.000^***^
E/A ratio	1.96 ± 0.89	1.94 ± 0.46	NS
E/e’ ratio	13.04 (10.00, 16.51)	9.34 (8.27, 10.00)	0.000^***^
**LA strain**			
LASr (%)	17.42 (9.99,30.08)	40.02 (31.88,50.33)	0.000^***^
LAScd (%)	-19.26 (-22.46,-12.65)	-34.82 (-40.46,-29.03)	0.000^***^
LASc (%)	0.60 (-8.08,3.56)	-6.60 (-9.37,-0.74)	NS
**TMAD**			
TMAD AP2 Midpt%	5.82 (0.94,7.64)	4.84 (2.61,6.68)	NS
TMAD AP2 Midpt (mm)	1.15 (0.27,1.69)	1.13 (0.59,1.46)	NS
TMAD AP4 Midpt%	4.06 (1.53,6.42)	3.63 (2.49,6.21)	NS
TMAD AP4 Midpt (mm)	0.91 (0.40,1.75)	1.03 (0.64,1.48)	NS

* indicates P value <0.05

*** indicates P value <0.001

### Echocardiographic parameters of the DCM patients across the timepoints

**Fig. 3:**
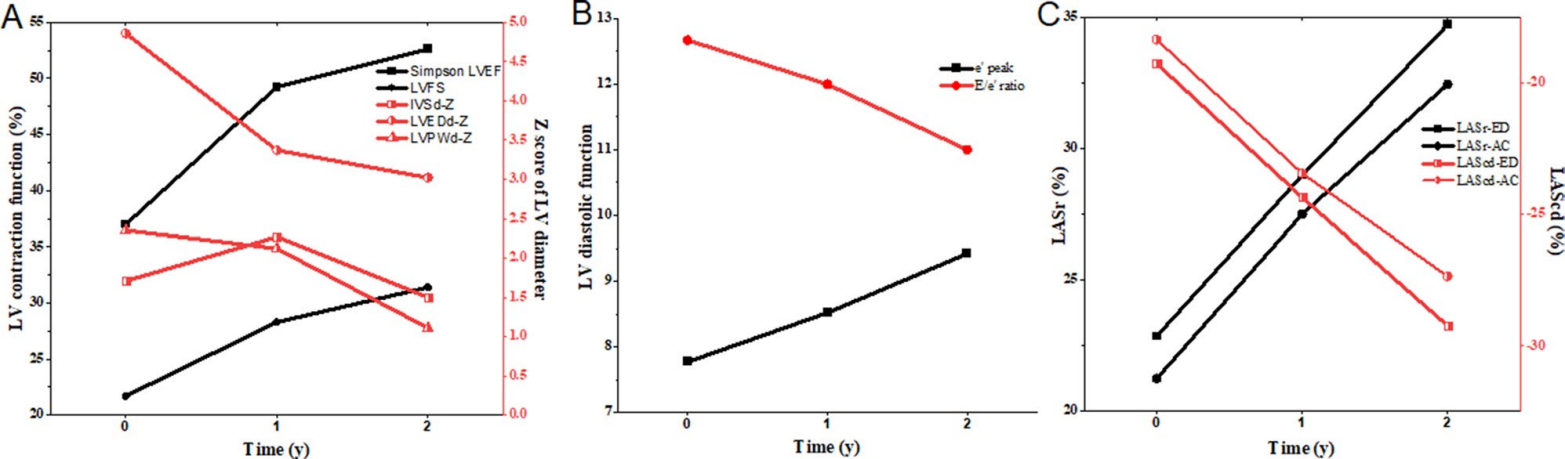
LV functional and LA strain parameters in DCM patients across the timepoints. The (**A**) LV systolic function and diameter Z scores, (**B**) LV diastolic function, and (**C**) LA reservoir and conduit strain of the patients

Compared to Baseline, significantly higher LVEF, LVFS, e’ peak, LASr and LAScd were observed in both Periods 1 and 2. Among the three groups, the IVSd-Z, LVEDd-Z, LVPWd-Z, and E/e’ ratio were significantly lower (*P* < 0.05). But LA-Z, E peak, A peak, LASct, TMAD AP2 Midpt%, TMAD AP2 Midpt, TMAD AP4 Midpt and TMAD AP4 Midpt% were not significant compared with each other ( *P* > 0.05). (Table 3; Fig. 3).

**Table 3:** LV and LA function parameters of DCM patients across the timepoints

Variable	Baseline	Period 1	Period 2	*P* value
	(*n* = 33)	(*n* = 33)	(*n* = 33)	
**LV systolic function**				
LVEF(%)	37.00 ± 15.57	49.24 ± 11.71	52.61 ± 11.78	0.000^***^
LVFS(%)	21.70 ± 8.92	28.30 ± 6.84	31.39 ± 8.71	0.000^***^
IVSd-Z	1.71 ± 1.27	2.27 ± 1.35	1.50 ± 1.34	0.015^*^
LVEDd-Z	4.86 ± 2.23	3.37 ± 2.44	3.02 ± 2.29	0.004^**^
LVPWd-Z	2.35 ± 1.81	2.12 ± 1.88	1.11 ± 1.52	0.003^**^
LA-Z (range)	-0.20 (-0.90, 1.40)	-0.10 (-0.70, 0.60)	-0.10 (-1.20, 1.25)	NS
**LV diastolic function**				
E peak (cm/s) (range)	100 (80, 112.5)	100 (92, 120)	100 (99, 115)	NS
e’ peak (cm/s)	7.77 ± 2.36	8.52 ± 2.05	9.49 ± 2.38	0.001^**^
E/A ratio (range)	1.71 (1.38, 2.86)	1.95 (1.50, 2.40)	1.62 (1.25, 1.82)	NS
E/e’ ratio (range)	12.67 (10.00,16.60)	12.00 (10.94, 14.38)	11.00 (10.00, 13.17)	0.007^**^
**LA strain**				
LASr (%)	22.86 ± 15.73	28.98 ± 9.04	34.72 ± 14.79	0.005^**^
LAScd (%)	-19.27 ± 8.98	-24.36 ± 6.77	-29.24 ± 11.51	0.021^*^
LASct (%)	-2.34 (-8.92,3.17)	-4.09 (-8.54,-1.65)	-5.90 (-9.00,-2.09)	NS
**TMAD**				
TMAD AP2 Midpt%	3.78 (1.03,6.03)	2.24 (0.00,3.71)	3.59 (0.62,8.42)	NS
TMAD AP2 Midpt (mm)	1.28 (0.67,1.94)	0.95 (0.47,1.17)	1.51 (0.89,3.21)	NS
TMAD AP4 Midpt%	4.06 (1.53,6.42)	2.57 (0.40,3.47)	3.28 (1.42,6.60)	NS
TMAD AP4 Midpt (mm)	0.92 (0.64,1.07)	1.24 (0.67,2.05)	1.31 (0.57,2.30)	NS

* indicates P value <0.05

** indicates P value <0.01

*** indicates P value <0.001

### Echocardiographic parameters between patients with normal LVEF in Period 2 and Control Group 2

In Period 2, LVEF normalization was observed in 26 patients (Simpson LVEF ≥ 55%), 13 of whom were male and female, respectively. The mean age was 63 months, while the mean follow-up duration was 27 months. Among the 7 DCM patients whose LVEF remained under normal range, the etiology of 5 (71.4%) was genetic, while that of 2 (28.6%) was unknown.

A comparative analysis was conducted in patients with normal LVEF in Period 2 compared to Control group 2. In the two groups, indicators such as LVEF, LVFS, e’ peak, LAScd and GLS were decreased, while indicators such as IVSd-Z, LVEDd-Z, LVPWd-Z, and E/e’ ratio were increased; in addition, there was no significant difference in indicators such as LA-Z, E peak and LASr (*P* < 0.05). (Table 4).

**Table 4:** Echocardiographic features between patients with LVEF normalization and controls in Period 2

Variable	Patients	Control Group 2	*P* value
	(*n* = 26)	(*n* = 30)	
**LV systolic function**			
LVEF, %	56 (54, 62)	62 (60, 66)	0.000^***^
LVFS, %	34.00 (29.50, 37.00)	38.50 (36.00, 41.75)	0.000^***^
IVSd-Z	1.30 (0.30, 2.65)	0.15 (-0.48, 1.58)	0.006^**^
LVEDd-Z	2.40 (1.50, 3.75)	0.15 (-0.65, 0.78)	0.000^***^
LVPWd-Z	0.91 ± 1.53	0.25 ±0.84	0.048^*^
LA-Z	-0.24 ± 1.35	-0.81 ± 0.89	NS
GLS, %	-14.80(-16.00, -14.00)	-22.20(-23.30, -21.70)	0.000^***^
**LV diastolic function**			
e’ peak, cm/s	10.00 (9.00, 11.00)	12.00 (11.20, 12.88)	0.000^***^
E peak, cm/s	100.00 (99.00, 110.00)	110.00 (100.00, 118.00)	NS
E/A	1.94 (1.67, 2.84)	2.14 (1.85, 2.95)	NS
E/e’	12.24 (10.20, 15.35)	10.00 (9.20, 11.80)	0.003^**^
**LA strain**			
LASr, %	38.12 ± 12.35	44.40 ± 14.56	NS
LAScd, %	-31.76 ± 9.95	-35.15 ± 10.62	0.018^*^
LASct, %	-6.36 ± 5.73	-5.71 ± 6.55	NS

* indicates P value <0.05

** indicates P value <0.01

*** indicates P value <0.001

### Comparison of the isolated DCM and DCM associated with LV hypertrabeculation subgroup across the timepoints

At baseline, significantly higher LVEF, LVFS, LASr, LAScd, and GLS were observed in DCM associated with LV hypertrabeculation patients compared to isolated DCM patients (*P <* 0.05). (Table 5).

**Table 5:** Comparison of isolated DCM and DCM associated with LV hypertrabeculation subgroup across the timepoints

Variable	Period	Isolated DCM (*n* = 19)	DCM associated with LV hypertrabeculation (*n* = 14)	*P* value
**LV function**				
LVEF, %	Baseline	29.00 (25.25, 38.75)	53.00 (39.50, 58.00)	0.003^**^
	Period 1	44.00 (36.75, 60.50)	59.00 (51.00, 60.00)	0.033^*^
	Period 2	55.00 (45.75, 61.50)	56.50 (54.00, 64.50)	NS
LVFS, %	Baseline	18.00 (13.75, 21.25)	30.00 (23.50, 34.75)	0.003^**^
	Period 1	27.50 (18.75, 33.50)	33.50 (29.00, 34.75)	0.050
	Period 2	29.00 (22.75, 35.75)	35.00 (31.00, 37.25)	NS
IVSd-Z	Baseline	2.25 (0.85, 3.50)	0.65 (-0.13, 1.43)	0.017^*^
	Period 1	2.30 (1.28, 3.45)	2.00 (0.85, 2.40)	NS
	Period 2	2.20 (0.60, 2.98)	1.00 (0.48, 2.55)	NS
LVEDd-Z	Baseline	5.40 (4.43, 7.63)	2.70 (2.35, 4.33)	0.002^**^
	Period 1	4.20 (1.40, 7.28)	2.56 (1.80, 2.30)	NS
	Period 2	2.70 (0.58, 4.88)	2.50 (1.68, 4.13)	NS
GLS, %	Baseline	-7.20 (-8.65, -5.95)	-13.30 (-14.05, -11.85)	0.011^*^
	Period 1	-12.33 (-13.93, -7.17)	-11.73 (-13.03, -10.53)	NS
	Period 2	-14.40 (-15.35, -8.75)	-15.30 (-16.08, -13.90)	NS
**LA strain**				
LASr, %	Baseline	12.89 (9.88, 25.85)	30.95 (19.36, 38.57)	0.019^*^
	Period 1	26.46 (18.72, 33.81)	32.99 (25.63, 41.23)	NS
	Period 2	31.66 (27.33, 36.62)	42.84 (24.01, 51.25)	NS
LAScd, %	Baseline	-15.80 (-21.14, -9.46)	-21.27 (-30.97, -18.58)	0.035^*^
	Period 1	-22.21 (-27.06, -17.81)	-26.76 (-33.56, -21.27)	NS
	Period 2	-27.31 (-29.69, -24.51)	-33.90 (-42.15, -22.47)	NS

* indicates P value <0.05

** indicates P value <0.01

### Correlation analyses

**Fig. 4:**
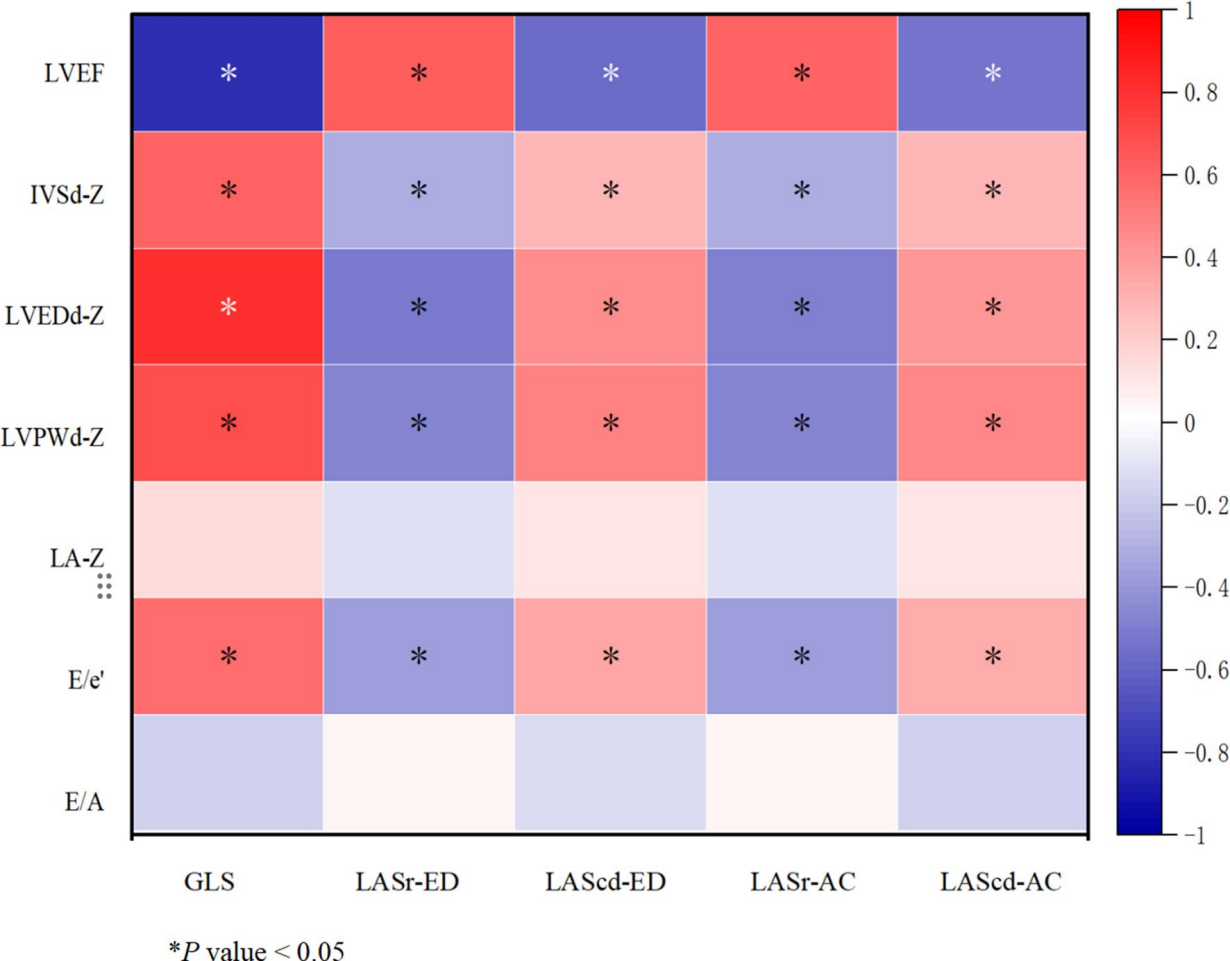
Correlation between left atrial strain and left ventricular function parameters of DCM patients. Red represents positive correlation, and blue represents negative correlation; the deeper and more saturated the color, the stronger the correlation between the two variables.*P value < 0.05. Abbreviations: GLS, Global longitudinal strain of the left ventricle; LASr-ED, longitudinal reservoir strain in end diastole; LAScd-ED, longitudinal conduit strain in end diastole; LASct-ED, longitudinal conduction strain in end diastole; LASr-AC, circumferential reservoir strain in peak systole; LAScd-AC, circumferential conduit strain in peak systole; LASct-AC, Circumferential conduction strain in peak; LVEF, left ventricular ejection fraction; TMAD AP4 Midpt%, mean displacement of the mitral annulus in the apical four-chamber view; e’, e’ of mitral valve pulse on doppler imaging; E/A, E peak- to-A peak ratio; and E/e’, E peak-to-e’ peak ratio

We conducted a correlation analysis for all patients with DCM. LA strain indices including LASr and LAScd significantly correlated with LVEF, LVFS, IVSd-Z, LVPWd-Z, LA-Z, e’ peak, and E/e’ ratio (all *P* < 0.05). LAScd negatively correlated with LVEF, LVFS, e’ peak, and positively correlated with IVSd-Z, LVEDd-Z, LVPWd-Z, LA-Z, and E/e’ ratio. GLS was negatively correlated with LVEF, and positively correlated with IVSd-Z, LVEDd-Z, LVPWd-Z, and E/e’ ratio. (Fig. 4).

## Discussion

In the DCM patients we have studied, impairment of LA conduit function and reservoir function were confirmed by 2D-STE prior to initiation of treatment. With the extension of standardized drug treatments, the LA function can significantly improve. However, even when their LV systolic function returns to normal level, their LA function remains below that of normal children.

In adult patients with DCM, LA strain is an independent and robust indicator for predicting adverse cardiovascular events, and its prognostic value is even superior to that of left ventricular ejection fraction (LVEF), left ventricular global longitudinal strain (LV GLS), and left atrial volume index (LAVI) [27]. A preliminary study published in 2024 enrolled 56 pediatric cardiomyopathy patients (including 28 cases of DCM), and the results showed that both LASr < 20% and LAScd ≥-12% were associated with decreased survival rate [28]. A study published in 2025 specifically explored the predictive value of LA strain for left ventricular reverse remodeling (LVRR) in DCM patients. Among 116 DCM patients, LAScd was an independent predictor of LVRR [29]. Another study involving 488 adult DCM patients drew similar conclusions, demonstrating that during a median follow-up of 6 years, LA conduit strain was a strong independent predictor of the primary endpoint (death, heart failure hospitalization, or life-threatening arrhythmia) [30]. However, in pediatric cardiomyopathy, especially pediatric DCM, relevant studies on LA strain are relatively scarce [1], and its prognostic significance as well as the differences from other cardiomyopathy subtypes still need to be systematically elaborated.

Recent studies have shown a yearly increase in the incidence of pediatric DCM [5]. The prognosis of DCM is considered poor, with complete recovery of cardiac function only reported in approximately 22% of cases following treatment [31–34]. In our study, the treatment was least effective in the genetic DCM patients, with only 50% of LVEF returning to normal. Subtypes of pediatric DCM exhibit distinct clinical characteristics and prognostic risks, and may have different response rates to current standard treatments [35].

Isolated DCM was observed to associate with larger LVDd and poorer LVEF, but the differences between them were reduced after treatment. LV hypertrabeculation is a ventricular phenotype identified by imaging studies, and has been considered a normal variant or a physiological response to conditions of increased preload or afterload [21]. Our findings may thereby be explained by the reduction in cardiac load, and thus the reversal of myocardial hypertrabeculation, as a result of treatment.

Compared to the Control Group1, DCM patients at baseline period showed reduced LASr and LAScd, with no significant difference in LASct and LA-Z. This indicates that at baseline, the LA reservoir and conduit functions were already impaired, while LA contraction function and LA size were still preserved. Furthermore, a follow-up analysis and a stratified analysis were performed on Group 2 patients with different subtypes as well as those with normalized LVEF, and the results showed that LAScd still was a sensitive indicator.

DCM often associates with extensive myocardial degeneration and interstitial fibrosis [36]. Such cellular changes may further influence the electrophysiological activity of myocardial cells, resulting in activation delay in the diseased segments, and thus exacerbate the impairment in LV function. The increased volume and pressure load on the LV result in heightened LV diastolic pressure, which hinder LA emptying at early diastole. This increase in blood volume retained at late diastole, in addition to the reduced conduit function of the LA, results in an enlarged and less compliant LA, which can eventually impair reservoir function as well.

Long-term treatment with standard cardiac medication such as inotropes, diuretics, and vasodilators can help to achieve a favorable prognosis. Indeed, heart failure medications have been shown to improve LA and LV function by prolonging diastolic filling time and increasing coronary artery perfusion, which effectively reverse myocardial remodeling, reduce volume load, and enhance myocardial segmental contraction [26, 37, 38]. But regarding the drug treatment regimens and dosages, we failed to address the potential impact of different medication combinations or doses on the results.

Acute atrial remodeling occurring within 1 week of exposure to stressors is often reversible [39, 40], while alterations which develop across a longer term are often irreversible [41–43]. The latter occurs in DCM, and can involve cellular processes such as cellular hypertrophy, increased collagen turnover [41, 44], changes in cellular signaling [45–48], and autonomic nervous system abnormalities [49]. Despite significant improvement observed in LASr and LAScd among our patients following long-term treatment, they remained lower when compared to their healthy counterparts. This may be due to the irreversible remodeling of the left atrium. Emphasis is thereby needed on not only the early and long-term management, but also the regular monitoring of LA conduit strain to improve the prognosis of DCM patients.

We found that the diastolic function indicators, for example e’, E/e’ *et al* and left ventricular GLS of children with DCM were different from those of healthy children, but all indicators were within the normal range. The current review indicated that the mean values of LA strains during the reservoir, conduit, and contraction phases were 47.3% (95% CI 42.5–52.1%), 32.8% (95% CI 27.8–37.8%), and 12% (95% CI 10.0-14.1%), respectively [50]. And the LA conduit strain we shown was − 29.83 ± 9.33 in patients with LVEF normalization of Period 2, which was lower than the above normal value. So the LA conduit strain maybe a potential and effective indicator to assess cardiac function in DCM patients.

TMAD is attained by tracking the motion of the mitral annulus relative to the apex of either the atrium or the ventricle. However, no significant differences were observed in all TMAD parameters between the patient and control groups of our study. This may be related to the lower amplitude of longitudinal motion of the myocardium in DCM patients, rendering TMAD an ineffective measure of LA function in such patients. Moreover, the measurement of TMAD is highly load-dependent and susceptible to interference from geometric factors [51].

Factors such as hypertension [52], atrial fibrillation [53], obesity [54], aging [55], genetics [56], inflammation [57] and gender [58] can affect LA function. However, whether there are specific pathophysiological characteristics and functional change patterns of LA under different etiologies remains insufficiently studied, and large-scale studies are still needed for further investigation and validation.

Our study had several limitations. First, this was a single-center and retrospective study, with a relatively small patient cohort, and the follow-up period was short. A standard conventional four-chamber view of echocardiography was subtly different to a LA-focused view, which might affect LA size and LA borders’ accuracy. Application of the updated diagnostic criteria for LV hypertrabeculation resulted in discontinuous cohort studies. Large-sample prospective studies or randomized controlled trials with long-term follow-ups are thereby warranted for the verification of our results.

## Conclusions

Following long-term standardized medication treatment, children with DCM exhibited significant improvements in left atrial reservoir and conduit functions. LAScd carries the potential as a sensitive imaging modality for LA strain in pediatric DCM.

## Supplementary Information

Below is the link to the electronic supplementary material.

Supplementary Material 1

## Data Availability

The authors confirm that the data supporting the findings of this study are available within the article.

## Abbreviations

2D-STE: Two-dimensional speckle tracking echocardiography
TMAD: Tissue mitral annular displacement
DCM: Dilated cardiomyopathy
HCM: Hypertrophic cardiomyopathy
RCM: Restrictive cardiomyopathy
NDLVC: Non-dilated left ventricular cardiomyopathy
ARVC: Arrhythmogenic right ventricular cardiomyopathy
NC/C: Non-compaction/Compaction
HR: Heart rate
LVEDd-Z: Left ventricular end diastolic internal dimension Z score
LA-Z: Left atrial internal diameter Z score
LVPWd-Z: Diastolic left ventricular posterior wall thickness Z score
IVSd-Z: Diastolic interventricular septal thickness Z score
LVEF: Left ventricular ejection fraction
LVFS: Left ventricular fractional shortening
E peak: Mitral valve peak flow velocity in early diastole
A peak: Mitral valve peak flow velocity in late diastole
e’ peak: Mitral annular velocity in early diastole
E/e’: E peak-to-e’ peak ratio
E/A: E peak-to-A peak ratio
LASr: Left atrial reservoir strain
LAScd: Left atrial conduit strain
LASct: Left atrial contraction strain
TMAD AP4 Midpt: Mean displacement rate of the mitral annulus in the apical four-chamber view
TMAD AP4 Midpt%: Mean displacement rate of the mitral annulus in the apical four-chamber view
TMAD AP2 Midpt: Mean displacement of the mitral annulus in the two-chamber view
TMAD AP2 Midpt%: Mean displacement rate of the mitral annulus in the two-chamber view
GLS: Global longitudinal strain of the left ventricle

## Declarations

### Disclaimer

The views expressed in the submitted article are our own and not an official position of the institution or funder.
