# Supplementary material for: Left atrial strain on 2D-STE in pediatric dilated cardiomyopathy
Source: Echo Res Pract. 2026 Jul 6;13:24. doi: 10.1186/s44156-026-00126-2 (PMC13335339; doi:10.1186/s44156-026-00126-2)
Supplement: Supplementary file 1 — Supplementary Material 1 [file 44156_2026_126_MOESM1_ESM.docx]

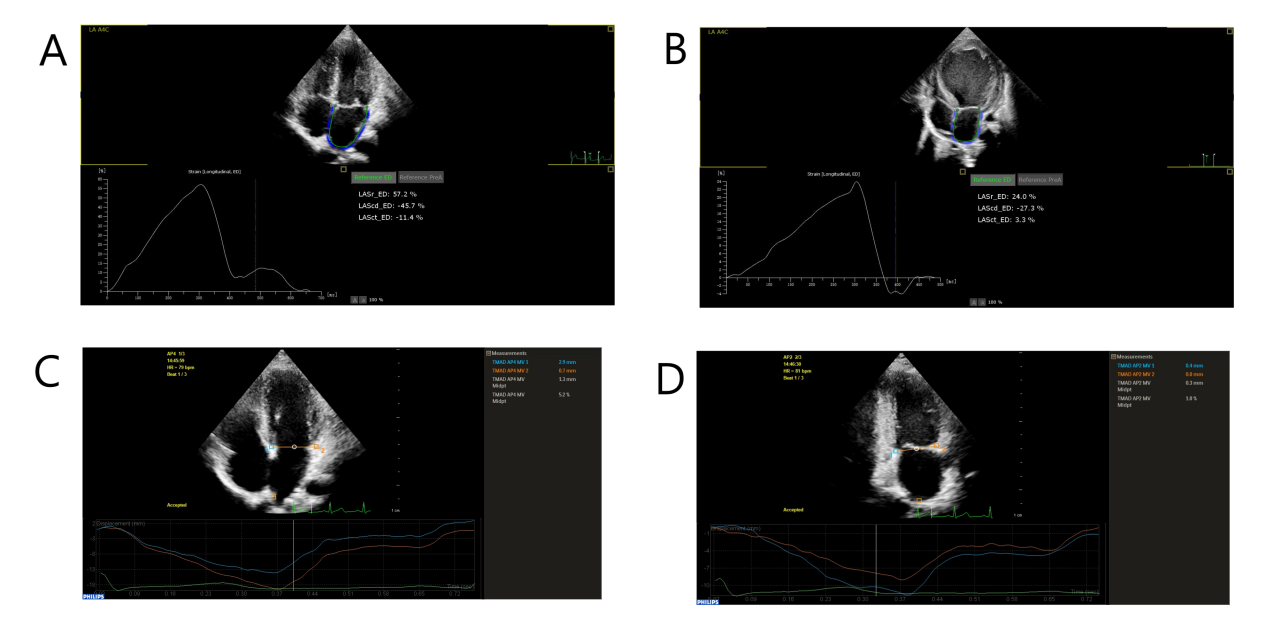


**Supplemental Figure 1. Left atrial function analysis.** The AutoStrain technique used during LA strain analysis in (A) a normal subject and (B) a patient. Evaluation of TMAD using (C) the apical four-chamber view and (D) the apical two-chamber view.


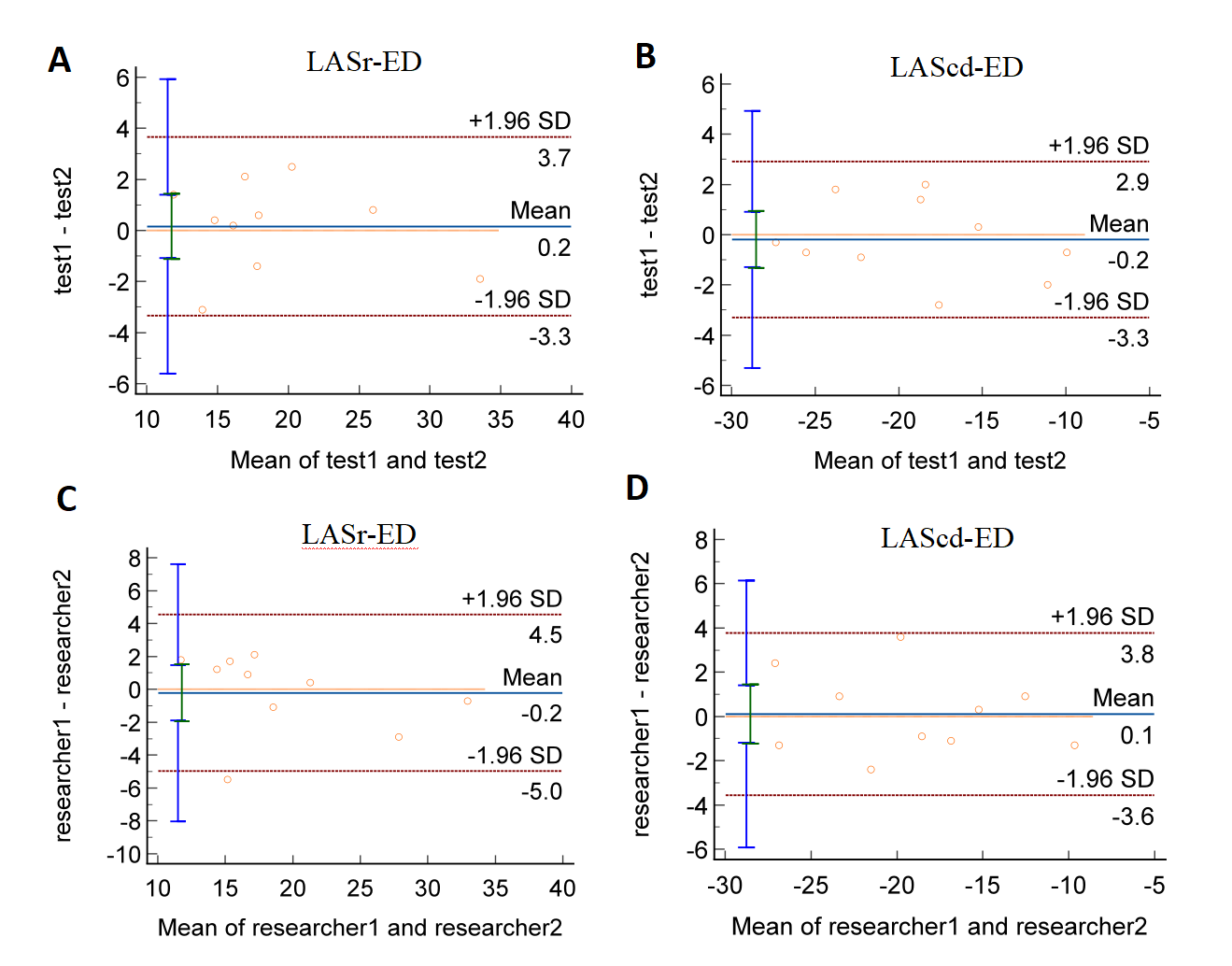


**Supplemental Figure 2. Bland-Altman analysis of inter-and intra-observer variability for LASr-ED and LAScd-ED.** Blue lines represent bias, and red dotted lines represent 95% limits of agreement for measurements performed in 10 patients.

**Supplemental Table 1 Common drugs and dosages used for treatment of patients**

| **Drug** | **Regimen** |
| --- | --- |
| **Captopril**  **Enalapril**  **Metoprolol**  **Carvedilol**  **Furosemide**  **Spironolactone**  **Digoxin**  **Milrinone**  **Sodium creatine phosphate**  **Coenzyme Q10**  **Levocarnitine**  **Fructose 1,6-diphosphate**  **Immunoglobulin**  **Hormones** | Oral: initial dose 0.15 mg/kg every 8–12 h; may be increased to a maximum dose of 6 mg/(kg.d) according to clinical efficacy  Oral: initial dose 0.1 mg/(kg.d),every 12 h; maximum dose 0.1 mg/(kg.d)  Oral: initial dose 0.10–0.25 mg/(kg.d), maximum dose 2 mg/(kg.d), total dose <100 mg/d  Oral: initial dose 0.10 mg/(kg.d), maximum dose 0.3–1.0m g/(kg.d), total dose <100 mg/d  Oral or intravenous injection: 0.5–2.0 mg/kg per dose, every 6–24 h; maximum dose 6 mg/(kg.h)  Oral: 1–3 mg/(kg.d), 2–4 times/d, maximum dose is 4–6 mg/(kg.d), total dose <100 mg/d  Digoxin dose (saturation): oral dose is 0.01–0.02 mg/kg for preterm infants, 0.02–0.03 mg/kg for full-term infants, 0.03–0.04 mg/kg for age <2 years, 0.02–0.03 mg/kg for age >2 years; intravenous dose is 75% of oral dose  Digitalis: give 1/2 of the digitalis dose in the first dose and the rest in two doses at intervals of 6–8 h; start maintenance dose 12 h after digitalis (maintenance dose is given daily at 25% of the digitalis dose in two doses)  Intravenous loading dose: 25–75 µg/kg, intravenous injection time >10 min; followed by 0.25–1.0 µg/(kg.min) intravenous drip maintenance; general dosing time is 7–10 d  Intravenous drip: infants and children 0.5 g each time, 1–2 times/d, older children 1.0 g/d each time, 1–2 times/d  Oral: 5–10 mg/(kg.d)  Oral or intravenous drip: 50–100 mg/(kg.d)  Intravenous drip: 50–150 mg/kg each time, once-daily; Oral: 0.5–1.0 g each time, 2–3 times/d  Intravenous injection: total 1–2 g/kg, divided over 3–5 days  0.5–2 mg/kg/d, divided into 1–3 doses |

**Supplemental Table 2 Repeatability and reproducibility of LASr and LAScd**

|  | Mean ± SD | Mean ± SD | Bias | 95% confidience  interval (bias) | P | 95% limit of agreement |
| --- | --- | --- | --- | --- | --- | --- |
| Inter-observer variability |  |  |  |  |  |  |
| LASr | 19.00 ± 6.32 | 18.84 ± 6.64 | 0.16 | -1.12 to 1.44 | 0.78 | -3.34 to 3.66 |
| LAScd | -19.09 ± 5.68 | -18.90 ± 6.10 | -0.19 | -1.32 to 0.94 | 0.71 | -3.30 to 2.92 |
| Intra-observer variability |  |  |  |  |  |  |
| LASr | 19.00 ± 6.32 | 19.21 ± 7.04 | -0.21 | -1.94 to 1.52 | 0.79 | -4.96 to 4.54 |
| LAScd | -19.09 ± 5.68 | -19.20 ± 6.02 | 0.11 | -1.23 to 1.45 | 0.86 | -3.56 to 3.78 |
